# Vestibular Loss and Cerebellar Ataxia: A Practical Approach

**DOI:** 10.1097/AUD.0000000000001708

**Published:** 2025-08-12

**Authors:** Lucia Joffily, Syuzanna Simonyan, Paola Giunti, Alexander Andrea Tarnutzer, Nehzat Koohi, Diego Kaski

**Affiliations:** 1 Department of Clinical and Movement Neurosciences, University College London, Queen Square Institute of Neurology, London, United Kingdom; 2 ENT Department, Universidade Federal do Estado do Rio de Janeiro, Rio de Janeiro, Brazil; 3 Neurology Department, Universidade Federal do Rio de Janeiro, Rio de Janeiro, Brazil; 4 Yerevan State Medical University after Mkhitar Heratsi, Yerevan, Armenia; 5 Ataxia Centre, Department of Clinical and Movement Neurosciences, UCL Queen Square Institute of Neurology, University College London, London, United Kingdom; 6 Department of Neurogenetics, National Hospital for Neurology and Neurosurgery, University College London Hospitals NHS Foundation Trust, London, United Kingdom; 7 Department of Neurology, Cantonal Hospital of Baden, Baden, Switzerland; 8 Faculty of Medicine, University of Zurich, Zurich, Switzerland; 9 These authors are joint senior authors.

**Keywords:** CANVAS, Cerebellar ataxia, Differential diagnosis, Spinocerebellar ataxias, Stroke, Thiamine deficiency, Vestibular loss, Vestibulo-ocular reflex

## Abstract

Cerebellar ataxia has been the remit of neurology but increased awareness of genetic disorders affecting both cerebellar and vestibular function has led to a rise in their reported prevalence. It is not uncommon for such patients to be seen in otolaryngology clinics. This review explores the underlying etiologies of patients presenting with vestibular loss accompanied by signs of cerebellar ataxia and provides a practical approach to diagnosis. We provide a comprehensive overview of common conditions that can manifest with both vestibular dysfunction and cerebellar ataxia, acutely or chronically, including cerebellar strokes, thiamine deficiency, and neurodegenerative diseases such as spinocerebellar ataxias. The article explores key diagnostic approaches, including clinical examination, neuroimaging, and specialized vestibular testing, to aid in distinguishing these conditions. By focusing on practical aspects of diagnosis, we offer otolaryngology specialists an essential tool for more accurate identification and management of patients, aiming to improve patient care. We emphasize the importance of a systematic, multidisciplinary approach to managing complex vestibular cases.

## INTRODUCTION

Most individuals with complaints of dizziness or balance issues will be referred to otolaryngology, audiovestibular medicine, or neurology. Otolaryngologists may focus on peripheral (inner ear) vestibular factors when evaluating a dizzy patient, while neurologists naturally prioritize the neurological features. However, many patients present with combined impairments in multiple balance-related structures, often involving vestibular and cerebellar systems (Barsottini et al. 2019; Borsche et al. 2024). Growing recognition of genetic disorders affecting cerebellar and vestibular functions has made them less rare and highlighted the need for otolaryngologists to have greater awareness of these—traditionally neurological—conditions. Otolaryngologists should suspect vestibular conditions linked to cerebellar dysfunction in patients with evidence of vestibular loss accompanied by ocular motor or gait cerebellar signs. Such an approach also needs to harness advances in neuroimaging and audiovestibular assessment tools.

This interplay between vestibular and cerebellar systems is complex, and their simultaneous dysfunction can lead to unique diagnostic challenges. From a symptomatic perspective, both can present with unsteadiness, dizziness, and visual disturbance (oscillopsia). Here, we first explore the mechanisms underlying vestibular and cerebellar impairments and their clinical implications. We specifically focus on the differential diagnosis of patients with vestibular loss and concurrent signs of cerebellar ataxia and provide an overview of the common conditions.

### Signs of Vestibular and Cerebellar Impairment

The angular vestibular ocular reflex (aVOR) maintains gaze stability during head rotation by generating eye movements opposite to the head movement (Riska et al. 2020). When vestibular loss occurs, aVOR gain is reduced, causing the eyes to move with the head, leading to visual blurring during ipsilesional head rotation (Riska et al. 2020). An aVOR deficit can be identified using the bedside head impulse test (HIT) in which the individual maintains the gaze on a target while the head is rapidly rotated; an abnormal response occurs when the eyes move off target, and a corrective saccade is necessary (Sealy 2014). Various complementary tests with different stimuli can be used to assess peripheral (inner ear) semicircular canal vestibular function, including mechanical stimuli (video HIT [vHIT] and rotatory chair) and thermal stimuli (caloric test), and acoustic or vibration stimuli for the otolithic organs (Striteska et al. 2021).

**Video 1.** 

**Video 2.** 

**Video 3.** 

**Fig. 1.**
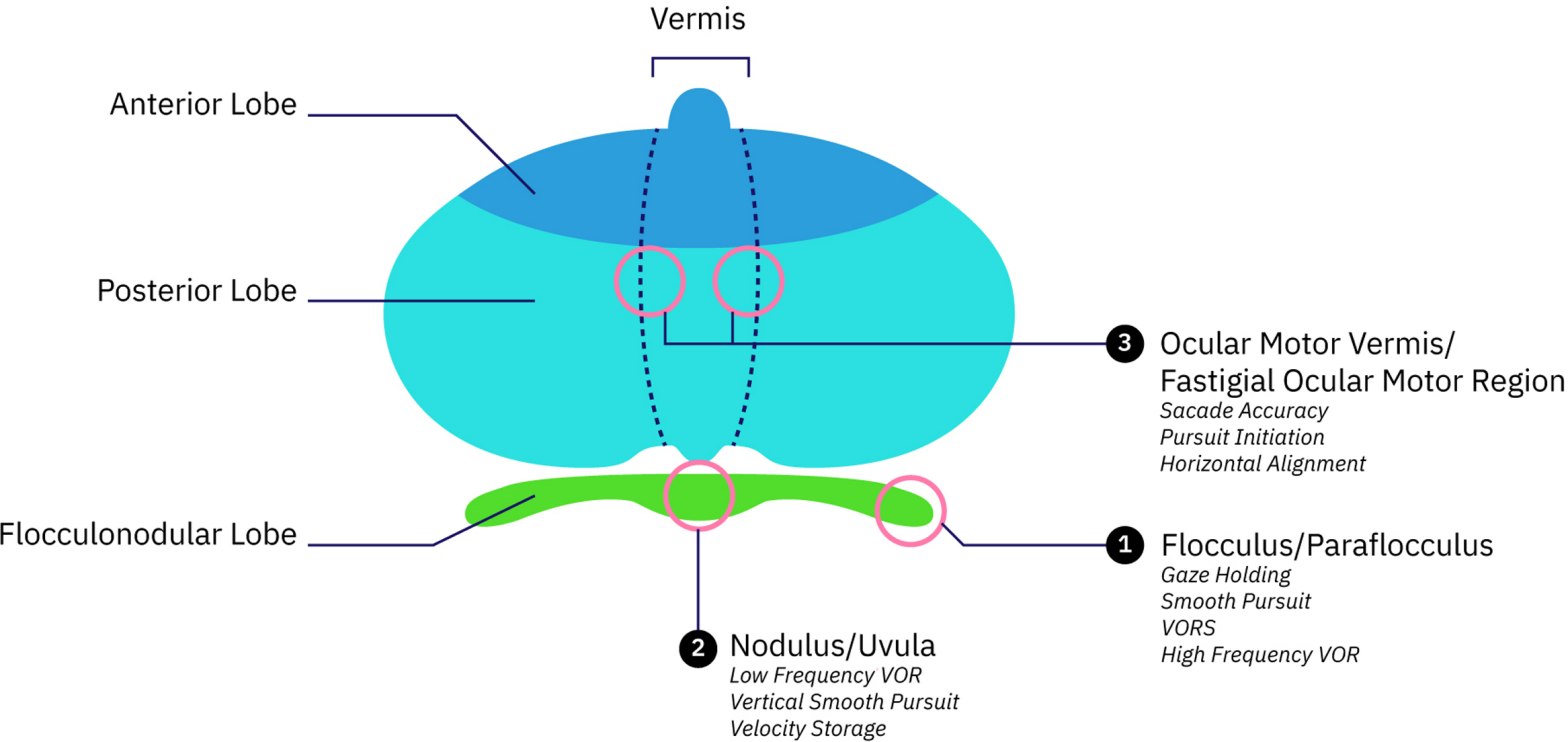
Anatomical cerebellar structures involved in eye movement control. VOR indicates vestibular ocular reflex; VORS, VOR suppression.

The cerebellum, responsible for coordinating voluntary movements, maintaining balance, controlling eye movements, and non-motor functions including cognition and mood, is impaired in cerebellar ataxia. The motor features are characterized by incoordination without muscular weakness or sensory loss (Radmard et al. 2023). Vertigo, dizziness, and imbalance are associated with lesions in the vestibulocerebellar, vestibulospinal, or cerebellar ocular motor systems (Bodranghien et al. 2016). There are three key areas in the cerebellum involved in eye movement control (Fig. 1): (1) the flocculus/paraflocculus (tonsil) complex, responsible for high-frequency vestibular responses, pursuit maintenance, and steady gaze holding; (2) the nodulus/ventral uvula, which handles low-frequency, sustained vestibular responses; and (3) the dorsal vermis/posterior fastigial nucleus, primarily involved in the accuracy of saccades (Shemesh & Zee 2019). Ocular instability, nystagmus, impaired saccadic (hypermetric [Video_1, Supplemental Digital Content, https://links.lww.com/EANDH/B743] or hypometric saccade), disrupted smooth pursuit, ocular misalignment, impaired VOR and impaired VOR suppression are at the core oculomotor cerebellar deficits (Bodranghien et al. 2016). Note that the cerebellum can be considered an extension of the vestibular nuclei due to its shared role in sensorimotor integration, direct anatomical connections, and functional refinement of vestibular reflexes. This is why many of the “central vestibular” eye movement abnormalities (i.e., such as those recorded during video-oculography) are in fact mostly cerebellar in origin.

Furthermore, the cerebellum plays a role in vestibular compensation (Shemesh & Zee 2019). Thus, the presence of cerebellar pathology can both induce a balance deficit but also exacerbate any imbalance from an existing vestibular pathology. When either the vestibular or cerebellar function is impaired in isolation, visually guided stabilizing eye movements, the aVOR and pursuit (along with the optokinetic reflex), respectively, compensate for maintaining gaze during low-frequency head movements. When both systems are impaired, this compensation is no longer effective, resulting in clinically observable catch-up saccades during visually enhanced vestibular ocular reflex (VVOR) (low-frequency head movements test [Video_2, Supplemental Digital Content, https://links.lww.com/EANDH/B744]) (Dupré et al. 2021). This can be a useful clinical sign when both vestibular and cerebellar involvement are suspected.

### Vestibular Loss and Cerebellar Ataxia Differential Diagnoses

There are several disorders that can present with combined cerebellar and vestibular lesions, such as stroke, cerebellopontine angle (CPA) tumor, thiamine (B1) deficiency, multiple sclerosis (MS), and various hereditary cerebellar ataxias (Choi et al. 2016; Chen & Halmagyi 2018; Kattah 2018). We will not extensively discuss some rarer etiologies identified in the literature, such as Gaucher disease, phenytoin toxicity, superficial siderosis of the central nervous system, paraneoplastic syndromes, and bovine spongiform encephalopathy given the practical nature of this review (but see Kattah 2017; Chen & Halmagyi 2018). Anterior inferior cerebellar artery (AICA) stroke and CPA tumors, which account for over 70% of cases, are the primary causes of disorders involving both peripheral and central vestibular lesions (Choi et al. 2016; Chen & Halmagyi 2018). In the following paragraph, we present the key aspects of the main differential diagnoses to consider (Table 1).

**TABLE 1.** Key clinical features of the common disorders affecting vestibular and cerebellar structures

Condition	Key Aspects
Acute	
B1 deficiency	The triad is encephalopathy, ataxia, and ophthalmoplegia (Chen et al. 2014). An acute vestibular syndrome can be present with bilateral horizontal canal VOR loss (on vHIT) (Choi et al. 2007). Ocular motor abnormalities include vertical nystagmus, horizontal gaze-evoked nystagmus, central positional nystagmus, internuclear ophthalmoplegia, and gaze palsies and may precede ataxia and encephalopathy (Choi et al. 2016). Some cases respond rapidly to a parenteral thiamine loading dose (Choi et al. 2022).
Stroke (AICA)	Common symptoms are acute-onset vertigo, vomiting, ipsilateral hemiataxia, Horner’s, and cerebellar ataxia (Clément et al. 2024). HINTS (Head Impulse, Nystagmus, and Test of Skew) describing a normal head impulse, direction-changing nystagmus, or skew deviation suggests stroke; HINTS+ (which adds a bedside hearing test) with a new unilateral hearing loss is a red flag for stroke in acute vestibular syndrome. Ipsilateral VOR deficit in bedside HIT or vHIT has been described (Clément et al. 2024). In floccular stroke, vHIT findings include both ipsilateral horizontal and vertical VOR deficits (vertical sparing is possible) and contralateral horizontal canal deficit (Burk et al. 1999). Catch-up saccades are of lower velocity compared with those seen in vestibular neuritis (Coarelli et al. 2023).
Chronic	
CANVAS	Autosomal recessive, onset age ±50 y/o (Cortese et al. 2019). Slowly progressive gait ataxia, falls, oscillopsia, dysarthria, sensory symptoms (tingling, numbness), and chronic cough (Bronstein et al. 1991). Sensory neuronopathy is present in most cases (Cortese et al. 2019). Severe bilateral VOR gain reductions (vHIT) in both vertical and horizontal canals (Donato et al. 2012; Cortese et al. 2020). Dysmetric saccades and broken pursuit have been described, together with impairment of the VVOR (Video_2, Supplemental Digital Content, https://links.lww.com/EANDH/B744) (Marrie et al. 2013). The vestibular areflexia is thought to be due to ganglionopathy (Lebre & Brice 2003).
SCA27B/*FGF14* gene	Autosomal dominant, late-onset, slowly progressive pan-cerebellar syndrome (Szmulewicz et al. 2014, 2016; Surmeli et al. 2022). Often accompanied by downbeat nystagmus, and episodic symptoms (gait ataxia, dysarthria, diplopia, oscillopsia, vertigo or dizziness, and appendicular ataxia). Often borderline abnormal bilateral VOR gain (vHIT), emerging later, and remaining mild, without progressing to severe vestibular areflexia (Guterman et al. 2016; Ng et al. 2021). The neuropathy impairment could be absent or mild (Guterman et al. 2016). The milder forms of both vestibular loss and neuropathy can help differentiate this condition from CANVAS.
FRDA	Autosomal recessive spinocerebellar ataxia, the disease onset ranging from 5 to 20 y/o (Martin 2012). Progressive ataxia, absence of tendon reflexes and weakness in the lower limbs, dysarthria, downbeat nystagmus, gaze-evoked nystagmus, VOR deficits (Ng et al. 2021), and auditory processing impairment (Guterman et al. 2016). Diabetes and hypertrophic cardiomyopathy are commonly present (Migliaccio et al. 2004). The younger age at disease onset, presence of auditory impairment, less profound VOR gain loss, and presence of frequent square-wave jerks can help to differentiate it from CANVAS.
MS	A chronic recurrent inflammation, demyelination, and gliosis occurring anywhere within the central nervous system, including the auditory (Kattah et al. 2019) and vestibular systems (Keita et al. 2022). An internuclear ophthalmoplegia, characterized by slowed adduction of the affected eye and abducting nystagmus, is a quite common eye movement disorder in MS. The most common audiovestibular is unilateral, moderate-profound, fluctuating, and sudden sensorineural hearing loss (Kattah et al. 2019). A pseudo-vestibular neuritis can result from an acute demyelinating brainstem lesion (Kim et al. 2023a). The VOR gain reduction can be associated with a greater MS-related disability (Kim et al. 2023a).
SCA	A heterogeneous group of autosomal dominant inherited ataxias, typically late-onset, progressive (Park et al. 2013). The triad of symptoms include gait ataxia (and incoordination), nystagmus (or other visual problems), and dysarthria (Pellerin et al. 2024). SCA3 (Machado-Joseph Disease) is the commonest subtype, presenting with gait imbalance, vestibular symptoms (mild to severe VOR gain reduction in vHIT), and hearing and speech difficulties (Ruano et al. 2014). Other SCAs to consider in patients with combined vestibular and cerebellar dysfunction include SCA1, SCA2, SCA6, and SCA7 (associated with retinal degeneration) (Park et al. 2013).
EA	It is a rare autosomal dominant channelopathies characterized by discrete attacks of ataxia of variable duration and frequency, also accompanied by interictal symptoms (Tu & Young 2004). It starts to manifest under 20 y/o (Pellerin et al. 2024). There are different subtypes; EA1 and EA2 are the most common (Tu & Young 2004). Interictal findings help distinguish it from vestibular migraine (Tu & Young 2004). There is some evidence of VOR impairment in EA2, even during interictal periods (Zeigelboim et al. 2017).

AICA, anterior inferior cerebellar artery; CANVAS, Cerebellar Ataxia with Neuropathy and Bilateral Vestibular Areflexia Syndrome; EA, episodic ataxia; FRDA, Friedreich’s ataxia; MS, multiple sclerosis; SCA, spinocerebellar ataxia; vHIT, video Head Impulse Test; VOR, vestibular ocular reflex; VVOR, visually enhanced vestibular ocular reflex.

### Stroke

Ischemic strokes are a common vascular cause of vestibular loss with cerebellar deficits (Kim et al. 2022). Although the posterior inferior cerebellar artery (PICA) is the most common affected territory in posterior circulation strokes, AICA strokes are the leading cause of sudden audiovestibular loss, but early brain magnetic resonance imaging (MRI) scans can be normal (even with diffusion weighted imaging, which is the gold standard for stroke identification) (Kim et al. 2022; Tarnutzer et al. 2023; Jaganathan et al. 2024). The prominent features of AICA infarction include acute-onset vertigo, vomiting, ipsilateral hemiataxia, Horner’s and cerebellar ataxia (Bery et al. 2022). A new-onset unilateral hearing loss in the context of acute vestibular syndrome indicating a red flag for AICA stroke (Bery et al. 2022). The AICA supplies the vestibulocochlear nerve, root entry zone, dorsolateral pons including the vestibular nuclei and flocculus, accounting for the audiovestibular and cerebellar signs (Chen & Halmagyi 2018). Dizziness from stroke shares a clinical phenotype with vestibular neuritis, and neuroimaging often fails to resolve this differential diagnosis, as non-contrast head computed tomography (CT) scans have low sensitivity for acute ischemic strokes in dizziness, with false-negative MRIs being common (Tarnutzer et al. 2023).

The vHIT presentations likely vary depending on the size and topography of the affected area, with an ipsilateral VOR deficit described in both bedside HIT and vHIT (Nham et al. 2022; Bery et al. 2022). AICA stroke on vHIT has also been observed as having two distinct presentations: (1) ipsilateral horizontal and verticals canals deficits, with contralateral horizontal canal deficit, and (2) bilateral horizontal canal deficit with vertical canal sparing (Chen & Halmagyi 2018). AICA stroke appear to produce a more symmetric bilateral gain reduction with smaller saccades on vHIT compared with vestibular neuritis (Chen et al. 2014). A case report described an isolated unilateral floccular stroke in the territory of the AICA, which caused bilateral horizontal low VOR gain during vHIT (high-frequency stimuli), with normal gain in vertical canals. In contrast, bithermal caloric tests (low-frequency stimuli) were normal, and the rotatory chair test (low-frequency stimuli) revealed discordant results with increased horizontal VOR gain (Park et al. 2013).

It is hypothesized that the flocculus modulates the VOR by inhibiting the horizontal VOR during low-frequency stimulation and facilitating it during high-frequency stimulation, but the sparing of vertical canals function is still unclear (Park et al. 2013; Chen & Halmagyi 2018). In addition, the smaller saccades observed on vHIT in AICA stroke compared with vestibular neuritis could be attributed to flocculus involvement, as it plays a role in modulating saccades (Chen et al. 2014).

An isolated heminodular stroke, affecting the nodulus with or without involvement of associated cerebellar structures supplied by the medial PICA, is rare but can mimic vestibular neuritis (Lee et al. 2023) A recent systematic review suggested that vHIT in PICA stroke is characterized by preserved VOR gain, no asymmetry, and reduced corrective saccade amplitude in the ipsilesional horizontal canals (Jaganathan et al. 2024).

### Thiamine (B1) Deficiency: Wernicke Encephalopathy

Vitamin B1 (thiamine) deficiency can cause acute cerebellar ataxia or subacute ataxia (Pedroso et al. 2019). Among patients with nutritional deficiency, thiamine (B1) deficiency is probably the leading cause and could be related to intractable vomiting and diarrhea from any etiology, such as chronic alcoholism, gastric bypass procedures, anorexia nervosa, fad diets, unbalanced parenteral hyper alimentation, cancer and chemotherapy, magnesium depletion, thyrotoxicosis, and renal disease (Kattah 2017).

The classic triad of Wernicke Encephalopathy are encephalopathy, ataxia, and ophthalmoplegia (Kattah 2018). Some patients present with an acute vestibular syndrome with selective, symmetrical impairment of horizontal VOR on vHIT, with sparing of vertical canals (Lee et al. 2018), without encephalopathy or ophthalmoplegia, which can occur in the early pre-encephalopathy stage of thiamine deficiency (Akdal et al. 2016; Lee et al. 2018).

Ocular motor abnormalities observed in Wernicke Encephalopathy include gaze-evoked nystagmus (Video_3, Supplemental Digital Content, https://links.lww.com/EANDH/B745), central positional nystagmus, abduction impairment, internuclear ophthalmoplegia, gaze palsies ranging from horizontal or vertical limitations to complete ophthalmoplegia (Choi et al. 2007). An up-beating nystagmus in primary gaze may transition to downbeat nystagmus within a few weeks and can persist over time (Kattah et al. 2019). This condition when associated with truncal ataxia could be related to paramedian tract neurons that project to the flocculus, suggesting a chronic cerebellar impairment (Kattah 2017). While some cases respond rapidly to a parenteral thiamine loading dose, normalization may take longer in others; particularly in late stages, like Korsakoff’s syndrome, the vestibular abnormalities often show only partial improvement (Kattah 2017).

### CPA Tumor

CPA tumors expand progressively and cause compression of the brainstem and cerebellum, resulting in variable lesion of anatomical substrates that are similarly affected by AICA stroke (Chen & Halmagyi 2018). The most common CPA lesion is vestibular schwannoma, with audiovestibular loss being slowly progressive, either bilateral or unilateral; the cerebellar signs vary widely and may include central positional nystagmus, gaze-evoked nystagmus, and Brun’s nystagmus (horizontal nystagmus with large and low frequency as the patient looks toward the side of the lesion [flocculus impairment], but a small and high-frequency primary position nystagmus that increases as the patient looks to the side opposite the lesion [vestibular lesion]) (Choi et al. 2016; Biswas et al. 2018).

### Multiple Sclerosis

MS is characterized by chronic inflammation, demyelination, and gliosis that are scattered and recurrent, with multiple plaques in the optic nerve, periventricular white matter, brain stem, and spinal cord; manifested as visual blurring, diplopia, sensory disturbance, ataxia, and weakness in one or more limbs (Tu & Young 2004). The reported prevalence of hearing loss in MS varies across the literature, ranging from 1 to 23.3% (MacMahon & El Refaie 2022), while dizziness is reported in 49 to 59% and vertigo occurs in 5% of patients (Marrie et al. 2013). The most common audiovestibular manifestation in MS patients was unilateral, moderate-profound, fluctuating, and sudden sensorineural hearing loss across all frequencies (250 to 8000 Hz) (MacMahon & El Refaie 2022). Vestibular symptoms may occur as an initial sign of the disease or develop during its progression (Surmeli et al. 2022). A pseudovestibular neuritis can result from an acute demyelinating lesion in the vestibular nucleus, presenting with unilateral vestibular loss on vHIT and accompanied by ataxia (Surmeli et al. 2022).

vHIT was performed on 37 MS patients with vertigo and/or imbalance symptoms, and the results were correlated with the level of MS-related disability (Grove et al. 2022). The data suggest that worse VOR function is associated with a greater MS-related disability (Grove et al. 2022). It has been proposed that in addition to MRI, audiovestibular tests (auditory brainstem response, vestibular-evoked myogenic potentials, caloric test, vHIT) are useful in monitoring MS patients who presented any neurotology symptoms (Tu & Young 2004; Surmeli et al. 2022).

### Hereditary Cerebellar Ataxias

Adult-onset hereditary cerebellar ataxias are progressive neurological disorders inherited in autosomal dominant, autosomal recessive, X-linked, or mitochondrial patterns. These conditions represent 10% of genetic diseases that affect the nervous system (Rudaks et al. 2024). The large number of causative genes and the clinical overlap between different genetic forms make diagnosis challenging (Rudaks et al. 2024.) In the following paragraph, we describe the hereditary cerebellar ataxias known to present with additional vestibular involvement.

#### Cerebellar Ataxia With Neuropathy and Bilateral Vestibular Areflexia Syndrome

Bronstein et al. (1991) were the first to describe vestibular loss associated with cerebellar ataxia, reporting two cases of multisystem atrophy affecting both the vestibular system and the cerebellum (Bronstein et al. 1991). It was noted that “the clinical pattern of those cases did not fit any single presently accepted entity,” as the patients lacked an acquired cause of cerebellar ataxia, and their vestibular deficits differed from those seen in hereditary cerebellar ataxias. Given the slow progression, absence of extrapyramidal signs, and lack of autonomic disorders, they proposed a novel syndrome termed cerebellar ataxia with bilateral vestibulopathy (Migliaccio et al. 2004). In 2011, retrospective data on 23 patients revealed that an axonal sensory neuropathy deficit is also a characteristic of this syndrome, which was termed “cerebellar ataxia with neuropathy and bilateral vestibular areflexia syndrome” (CANVAS) (Szmulewicz et al. 2011, 2016).

CANVAS is a neurodegenerative progressive late-onset ataxic condition, with an average onset age of 52 years old (Cortese et al. 2020). Although 16% of clinical cases suggest autosomal recessive inheritance, 55% of genetic cases are identified as sporadic (Szmulewicz et al. 2014; Cortese et al. 2020; Dupré et al. 2021). Biallelic intronic AAGGG repeat expansions in the replication factor C subunit 1 gene encoding a subunit of a DNA polymerase accessory protein have been identified as the cause of CANVAS (Cortese et al. 2019). A series of genetically confirmed cases showed that the syndrome involves cerebellar dysfunction, proprioceptive loss, and/or vestibular deficits, with the timing of onset of the different components varying, and a reported 10-yr delay in developing the full triad (Cortese et al. 2020). The full triad was present in 69% of cases, isolated neuronopathy in 15%, a combination of cerebellar ataxia and neuronopathy in 16%, and a combination of vestibular areflexia and neuronopathy in 6% (Cortese et al. 2020). There were no cases of vestibular areflexia and cerebellar ataxia occurring without neuronopathy, nor were there any cases of isolated cerebellar ataxia or vestibular areflexia (Cortese et al. 2020).

The neurological symptoms include postural imbalance (worse in darkness), dizziness, falls, sensory issues, or oscillopsia (see Supplemental Digital Content, https://links.lww.com/EANDH/B719, for case example and associated videos of the mild gait ataxia [Video 4, Supplemental Digital Content, https://links.lww.com/EANDH/B746], gaze-evoked and downbeat nystagmus [Video 5, Supplemental Digital Content, https://links.lww.com/EANDH/B747], bilaterally abnormal HIT [Video 6, Supplemental Digital Content, https://links.lww.com/EANDH/B748]). Oscillopsia during head movements is associated with bilaterally impaired VOR and can be easily observed in the HIT. Over time, dysarthria, dysphagia, and autonomic dysfunction may develop, with chronic cough often preceding neurological symptoms by decades (Dupré et al. 2021). Hearing remains unaffected (Dupré et al. 2021). Brain MRI shows cerebellar atrophy mainly affecting the anterior and dorsal vermis and the lateral cerebellum (Coarelli et al. 2023).

Clinical signs of cerebellar dysfunction in CANVAS include a combination of cerebellar dysarthria, appendicular ataxia, truncal ataxia, and cerebellar-related ocular motor deficits (dysmetric saccades with a normal velocity, smooth pursuit impaired showing square waves and broken pursuit) (Dupré et al. 2021). VOR gain reductions (vHIT) are typically observed in both vertical and horizontal canals and are often described as severe, although this depends on the disease stage (Borsche et al. 2024; Pellerin et al. 2024b). In addition, the combined deficit in vestibular and cerebellar ocular motor functions results in impairment of the VVOR (Video_2, Supplemental Digital Content, https://links.lww.com/EANDH/B744) (Petersen et al. 2013). The visually suppressed VOR (VORS) is “falsely normal” in CANVAS patients, due to absence of VOR (there being no VOR to suppress) (Dupré et al. 2021).

The disease progresses slowly, with 55% of patients requiring a walking stick after an average of 10 yr, and 25% needing a wheelchair after 15 yr of disease duration (Cortese et al. 2020). A post-mortem study showed significant loss of Purkinje cells (mainly in the vermis and lateral cerebellum), as well as Scarpa’s, trigeminal, and facial ganglion cells, but not spiral ganglion cells. The auditory nerve was unaffected, suggesting that vestibular areflexia is due to a ganglionopathy (Szmulewicz et al. 2011).

#### Friedreich Ataxia

Friedreich ataxia (FRDA) is an autosomal recessive spinocerebellar ataxia (SCA) caused by homozygous inheritance of FXN alleles with a trinucleotide GAA repeat expansion in intron 1 (Keita et al. 2022). It is the most common inherited ataxia in Europe and a slowly progressive neurodegenerative disease, with a progression rate that is neither constant nor linear (Fahey et al. 2008). Typically, onset of FRDA occurs between the ages of 5 and 20 yr of age, but it can present outside this range with some patients presenting as late as their 60s, depending on the number of repeat expansions (Keita et al. 2022).

FRDA can affect multiple areas of the central and peripheral nervous system, and as the disease progresses, individuals may become increasingly dependent on assistance with activities of daily living (Fahey et al. 2008). Progressive ataxia that initially affects the lower limbs and then the upper limbs, absence of tendon reflexes and weakness in the lower limbs, dysarthria, loss of distal deep sensitivity, and bilateral Babinski sign result from the disease’s progression in the brain, spinal cord, and peripheral nerves (Zeigelboim et al. 2017). Other common features include nystagmus (downbeat nystagmus, gaze-evoked nystagmus), optic atrophy, hearing difficulties particularly in the presence of background noise, atrophy in hands and distal lower limbs, scoliosis, pes cavus, and claw toes (Zeigelboim et al. 2017; Sohns et al. 2024). Diabetes and hypertrophic cardiomyopathy are frequently present (Fahey et al. 2008).

A recent systematic literature review on ocular motor and vestibular measurements in FRDA demonstrated presence of square-wave jerks (90/109), ocular flutter (21/43), impaired eccentric gaze holding (40/104), abnormal pursuit (81/93), increases in saccade latency (27/38), dysmetric saccades (71/93), and VOR deficits (39/48) (Sohns et al. 2024). Twenty-seven FRDA patients underwent audiological assessments, including pure-tone audiometry, otoacoustic emissions, and auditory brainstem response testing, revealing an association between genotype and the severity of auditory temporal and spatial processing deficits, which contributed to poorer speech perception (Koohi et al. 2021).

#### Spinocerebellar Ataxias

SCAs are autosomal dominant inherited ataxias, which constitute a heterogeneous group of typically late-onset, progressive, and often fatal neurodegenerative disorders, characterized by progressive cerebellar dysfunction, variably associated with other symptoms of the central and peripheral nervous systems (Bettencourt & Lima 2011). A recent systematic review found that the global prevalence of SCA is 3 per 100,000 individuals (Ruano et al. 2014). Almost 50 subtypes of SCAs have been identified; many SCAs are caused by CAG nucleotide repeat expansions that encode polyglutamine, and therefore, involve the toxic polyglutamine protein (Soong & Paulson 2007; Sullivan et al. 2019). SCA3 is the commonest subtype around the globe (Sullivan et al. 2019). The core triad of symptoms of SCAs includes gait ataxia and incoordination, nystagmus, other visual problems, and dysarthria, with patients also presenting additional features such as pyramidal and extrapyramidal signs, ophthalmoplegia, and cognitive impairment in specific SCAs (Sullivan et al. 2019). Neuroimaging studies in SCAs found atrophy of infratentorial structures, especially pons, medulla, and spinal cord to be a common feature (Barsottini et al. 2019).

##### Spinocerebellar ataxia type 1

Spinocerebellar ataxia type 1 (SCA1) was the first progressive autosomal dominant cerebellar ataxia to be genetically characterized; it is caused by an expanded CAG repeat in the coding region of the human ATXN1 gene (Donato et al. 2012). The age at onset of the disease can be extremely variable, ranging from 4 to 74 yr (Donato et al. 2012). The first symptoms of the disease are usually represented by loss of motor coordination, imbalance, and gait unsteadiness, which become evident in the fourth decade of life in most patients (Donato et al. 2012). The extracerebellar clinical presentation vary across pyramidal signs, dysphagia, oculomotor abnormalities (hypermetric saccades and nystagmus). As it progresses, nystagmus may disappear, saccades become slow, and an upward gaze palsy may develop; in advanced disease stages, horizontal gaze palsy impairs eye movements with the appearance of compensatory head movements (Donato et al. 2012). Peripheral nervous system involvement is found in at least half of the patients with SCA1 (Linnemann et al. 2016). In most SCA1 patients, MR imaging reveals global atrophy of the structures of the posterior fossa, consistent with the neuropathological finding of olivopontocerebellar atrophy (Donato et al. 2012; Linnemann et al. 2016). In a small study (n = 4), three patients manifest a reduced VOR gain (<0.77—vHIT) (Luis et al. 2016). Another study with 12 SCA1 patients found that 70% had a VOR gain (rotatory chair) below the normal limit, and 55.6% showed a reduced caloric response (Burk et al. 1999).

##### SCA type 3

SCA type 3, also known as Machado-Joseph disease, is an autosomal dominant neurodegenerative disease caused by an expansion of CAG trinucleotide repeats in the ATXN3 gene; it causes an aberrant protein that easily aggregates and deposits in the neurons of the cerebellum and brainstem (Elyoseph et al. 2024). The mutation causes the expression of aberrant protein with expanded polyglutamine stretch that easily aggregates and deposits in the neurons of the cerebellum and brainstem.

The age of onset is highly variable with a median of 40 yr (range 4 to 70 yr), and a mean survival time of 21 yr (Elyoseph et al. 2024). SCA3 primarily features a progressive cerebellar ataxia that also affects the brainstem, oculomotor system, and motor pathways; also including extrapyramidal syndromes (basal ganglia involvement), amyotrophy (brainstem and spinal cord involvement), and neuropathy (peripheral nervous system involvement) (Paulson 2012). It typically presents with gait imbalance, vestibular symptoms, and speech difficulties. As the disease advances, patients may experience nystagmus, slowed saccades, and ophthalmoplegia. In later stages, individuals may become wheelchair-bound and suffer from severe dysarthria and dysphagia. Other clinical presentations can include autonomic symptoms, facial and temporal atrophy, with dementia being uncommon but possible (Paulson 2012). Magnetic resonance imaging of the brain reveals atrophy of the brainstem and cerebellum (Paulson 2012). An audiological assessment was conducted on 12 SCA3 patients, with 33.3% showing abnormal audiometric test results and 58.3% exhibiting abnormal auditory brainstem responses (Zeigelboim et al. 2015).

VOR deficits are often present on SCA3, ranging from mild to severe (Luis et al. 2016; Kim et al. 2023). It has been proposed that the deficit in the horizontal VOR gain may serve as a neurophysiological biomarker for detecting the clinical onset, severity, and progression of SCA3; horizontal VOR gain measured by the vHIT was significantly low in almost all patients and inversely correlated with disease severity (Elyoseph et al. 2024). It has been assumed that VOR deficit gain on SCA3 is of central and not vestibular peripheral origin; the degeneration of the medial vestibular nucleus and nucleus prepositus hypoglossi could account for both central vestibular loss and gaze-evoked nystagmus (Gordon et al. 2014). Using vestibular-evoked myogenic potentials, researchers described that peripheral pathways emerging from the otoliths are also damaged in SCA3, with abnormal results found in 93% of 14 patients tested (Ribeiro et al. 2015; Barsottini et al. 2019).

##### Spinocerebellar ataxia type 6

Spinocerebellar ataxia type 6 (SCA6) is one of three allelic autosomal dominant disorders due to mutations of the CACNA1A gene, located on chromosome 19p13; the other two being episodic ataxia type 2 (EA2) and familial hemiplegic migraine (Tarnutzer et al. 2024). It is considered a late-onset disorder, with a mean age of onset in the fifth decade (Solodkin & Gomez 2012). Initial symptoms are gait unsteadiness, stumbling, and imbalance in about 90% of cases, rarely preceded for several years by symptoms of diplopia or episodic vertigo and vibratory, with proprioceptive sensory loss (Solodkin & Gomez 2012). Incoordination progress slowly, and eventually all patients have gait ataxia, upper limb incoordination, intention tremor, and dysarthria; lifespan is not shortened (Solodkin & Gomez 2012). MRI brain scans in SCA6 show essentially pure cerebellar atrophy (Solodkin & Gomez 2012). A systematic literature review in SCA6 patients of quantitative ocular motor and vestibular measurements, reported that the most frequently identified impairments included smooth pursuit, saccade metrics, gaze holding (gaze-evoked nystagmus and downbeat nystagmus), and high-frequency VOR deficit (Tarnutzer et al. 2024).

##### Spinocerebellar ataxia type 7

Spinocerebellar ataxia type 7 (SCA7) is a progressive autosomal dominant neurodegenerative disorder, caused by expansion of an unstable trinucleotide CAG repeat encoding a polyglutamine tract in the corresponding protein, ataxin-7 (Martin 2012). The clinical condition can develop during childhood up to the age of 60, with the disease progressing more rapidly in younger individuals (Lebre & Brice 2003). It affects primarily the cerebellum and the retina (pigmentary macular degeneration with color vision and visual acuity abnormalities), but also many other structures as the disease progresses (Lebre & Brice 2003). The association of cerebellar ataxia and dysarthria with pyramidal signs (increased reflexes and/or extensor plantar reflexes and/or lower limb spasticity), supranuclear ophthalmoplegia, slow saccades and decreased visual acuity is highly suggestive of SCA7 (Lebre & Brice 2003). Brain imaging shows marked atrophy in the cerebellum, particularly in the superior part of the vermis and the brainstem (Lebre & Brice 2003). Vestibular performance in SCA7 has rarely been studied; a recent study in 9 SCA7 patients impaired VOR gain on vHIT, particularly in the vertical canals (77.8%), greater for the anterior than the posterior canal (Kim et al. 2023). Although it has been suggested that visual acuity may not influence vHIT outcomes, it is not possible to rule out the impact of retinal degeneration on VOR impairments in SCA7 patients, requiring further investigation (Kim et al. 2023).

##### SCA27B/*FGF14* gene

SCA27B is an autosomal dominant disorder caused by a GAA repeat expansion in intron 1 of the fibroblast growth factor 14 (*FGF14*) gene, that has recently been recognized (2023) as one of the most common genetic forms of late-onset ataxia (Wilke et al. 2023; Clément et al. 2024; Pellerin et al. 2024b). It is a slowly progressive pan-cerebellar syndrome often accompanied by cerebellar oculomotor signs, such as downbeat nystagmus, and episodic symptoms.

The diagnosis of SCA27B is made by clinical evidence and the identification of a GAA expansion in intron 1 of *FGF14* (Clément et al. 2024). The phenotypic profile was described as: “A recognizable constellation of symptoms, which are recurrent, intermittent and discrete with clear onset and offset from the patient’s established baseline, and can appear unprovoked or be induced, for example, by alcohol or physical activity; characterized primarily by episodic cerebellar symptoms (gait ataxia, dysarthria, diplopia, oscillopsia, vertigo and/or dizziness or appendicular ataxia)” (Ashton et al. 2023). The majority of patients subsequently develop permanent ataxia after an average of 3 to 4 yr of disease (Clément et al. 2024).

The cerebellar sign on the examination comprises, gait impairment, broken-up smooth pursuit, saccadic dysmetria, and downbeat nystagmus (Wilke et al. 2023). These symptoms likely indicate involvement of the cerebellar flocculus and paraflocculus, with MRI revealing brain atrophy that primarily affects the cerebellar vermis and, to a lesser extent, the hemispheres (Clément et al. 2024). SCA27B ataxia have recently been shown to account for almost 50% of yet idiopathic downbeat nystagmus cases (Pellerin et al. 2024b), it is observed in up to 70% of 50 SCA patients (Ashton et al. 2023) and, as such, appears to be a particularly specific clinical manifestation of that specific ataxia. Regarding vestibular function, bilateral vestibulopathy (vHIT) is observed in 10 to 75% of individuals with SCA27B, emerging later in the disease and generally remaining mild, without progressing to severe vestibular areflexia (Borsche et al. 2024; Pellerin et al. 2024a). The later finding has clinical utility, as in CANVAS the loss of the VOR is more severe compared with SCA27B (Borsche et al. 2024).

Regarding the neuropathy impairment, while early studies suggested that polyneuropathy is not a core feature of SCA27B, subsequent research has shown that some patients may develop mild axonal sensory or sensorimotor polyneuropathy, and it differs from CANVAS, where the neuropathy is a hallmark of the disease (Ashton et al. 2023; Pellerin et al. 2024a). In addition, it was suggested that dysautonomia increased with duration while cognitive impairment remained infrequent, even in advanced stages (Wilke et al. 2023). Functional impairment progressed slowly, with 50% of patients using unilateral mobility aids after 8 yr (Wilke et al. 2023) and nystagmus and gait can improve with 3,4-diaminopyridine.

#### Mitochondrial Disease

Since mitochondria are essential organelles in all human cells, mitochondrial disease (MD) can affect all organs, involvement of the nervous system often occurs (Ng et al. 2021). Ataxia may be the dominant manifestation of MD, which includes pure cerebellar, sensory, or SCA (Hougaard et al. 2019). Vestibular dysfunction has been identified as an important manifestation of MD in adults (Hougaard et al. 2019). Many MDs were defined by a cluster of clinical features, such as mitochondrial myopathy, encephalopathy, lactic acidosis, stroke-like episode syndrome, and Kearns-Sayre syndrome (Ng et al. 2021). Although vestibular dysfunction has been rarely studied in mitochondrial myopathy, encephalopathy, lactic acidosis, and stroke-like episodes, some research using vHIT has shown bilateral abnormalities in the horizontal and posterior canals (Hougaard et al. 2019). Hearing impairment is an additional feature of MD, involving both the peripheral and central auditory systems (Koohi et al. 2024).

#### Episodic Ataxia

The term “episodic ataxia” (EA) refers to a small group of rare autosomal dominant channelopathies inherited disorders characterized by discrete attacks of ataxia of variable duration and frequency, often accompanied by other clinical symptoms, including in the interictal phase (Hassan 2023). EA is not typically associated with chronic deficits such as vestibular loss. However, we included it in this article because most EA patients experience both vertigo and ataxia, and some present with interictal symptoms. Further studies are needed to determine whether EA patients exhibit chronic vestibular deficits.

EA comprises some different subtypes, with EA1 and EA2 being the most common, caused by mutations in KCNA1 and CACNA1A, respectively (Hassan 2023). EA mostly manifests before age 20 yr, and it can be associated with other paroxysmal neurological disorders such as migraines, epilepsy, and dystonia (Sullivan et al. 2019). The key clinical feature that raises the diagnosis of EA is discrete attacks of incoordination with a clear onset and resolution of symptoms, which also distinguishes EA with progressive features from progressive ataxia with intermittent exacerbation; furthermore, the presence of interictal findings in most patients provides a helpful distinction with vestibular migraine (Hassan 2023).

EA1 typically begins in childhood, with an average onset at 7.8 yr of age. It is characterized by persistent myokymia in the face or limb muscles, detectable clinically or via electromyography. Attack lasts minutes and triggers include stress, startle, caffeine, alcohol, or sudden movement (Hassan 2023). The attacks consist of episodes of vertigo lasting minutes associated with diplopia, headache, stiffness of the body, and dysarthric speech (Hassan 2023). During an attack, gait impairment can range from mild dysfunction to complete inability to walk; furthermore, there may be comorbid cognitive disability or deafness (Hassan 2023). Most patients have normal cerebellar function between attacks and normal MRI brain imaging; however, longer disease duration is correlated with permanent cerebellar signs and cerebellar atrophy (Hassan 2023). Many patients do not seek treatment because attacks are brief and improve with age; a variety of antiseizure medications can diminish attacks, including carbamazepine, phenytoin, lamotrigine, and acetazolamine (Hassan 2023).

EA2 is the most frequent subtype of EA, usually begins in early childhood, most often before the age of 20, though symptoms may rarely first manifest in patients older than 50 yr (Gazquez & Lopez-Escamez 2011). It presents with attacks, lasting for several hours to days, of imbalance, vertigo, and ataxia which are provoked by physical exertion, emotional stress, or alcohol (Guterman et al. 2016). Ataxia and nystagmus may be present during the interictal phase (Gazquez & Lopez-Escamez 2011). The spells may be characterized by isolated ataxia or, as seen in our patient, a broader range of symptoms, often localizing to the brainstem; associated features include generalized and hemiplegic weakness, migraine, intellectual disability, dystonia, and seizures (Guterman et al. 2016). Therapeutically, acetazolamide responsiveness is a hallmark of the disease, with 50 to 75% of patients reporting improvement in episode severity and frequency, although some may not experience symptomatic benefit. Few studies have evaluated vestibular function during the interictal periods of EA (Guterman et al. 2016). A recent study assessed vestibular function (bithermal caloric tests, rotatory chair test, vHIT, and otolith function tests) in 17 EA2 patients. The results showed abnormal VOR responses in at least one semicircular canal with high acceleration and frequency head impulses (14/16, 88%), normal responses to low acceleration and frequency stimuli, and impairments in at least one otolith function test (13/16, 81%) in most patients. These findings suggest that vestibular impairments are common in EA2, even during interictal periods, which may indicate degeneration of the vestibulocerebellum or vestibular nuclei (Choi et al. 2022).

EA3 is a rare disorder characterized by recurrent attacks (lasting minutes to hours) of vestibular ataxia, vertigo, tinnitus, and headache, with interictal myokymia present in about half of the patients, and typically respond to acetazolamide (Gazquez & Lopez-Escamez 2011). EA4, or familial periodic vestibulocerebellar ataxia, is an autosomal dominant disorder marked by vertigo and ataxia episodes. Patients may have interictal nystagmus and mild ataxia, similar to EA2. Inability to suppress the VOR is the most consistent symptom (Gazquez & Lopez-Escamez 2011).

Finally, we present key considerations for investigating vestibular loss in cerebellar ataxia (Table 2) and recommend relevant tests for assessing vestibular loss associated with cerebellar ataxia (Table 3).

**TABLE 2.** Important clinical considerations when investigating vestibular loss in patients with cerebellar ataxia

Key Considerations for Investigating Vestibular Loss in Cerebellar Ataxia
• Age at onset • Family history • Acute vs. episodic vs. chronic presentation • Hearing loss • Peripheral sensory neuropathy • Vestibular loss pattern on vHIT

vHIT, video Head Impulse Test.

**TABLE 3.** Investigations for patients with vestibular loss and cerebellar ataxia

Tests to Consider for Vestibular Loss Associated With Cerebellar Ataxia
• Serological tests (blood count, electrolytes, liver and bone profile, thyroid function, vitamin B1, vitamin B12, vitamin E, autoimmune profile, paraneoplastic panel) • Genetic tests (SCA panel, RFC1, EA2, FXN, WGS [if available]) • MRI brain • Cerebrospinal fluid examination (autoimmune/inflammatory) • Nerve conduction study (hereditary ataxias with neuropathy) • PET scan (for suspected paraneoplastic syndrome) • Hearing assessment (pure-tone audiometry, brainstem responses) • Vestibular assessment (vHIT, caloric test, rotatory chair, VEMP)

EA2, episodic ataxia 2; FXN, frataxin FXN gene-Friedreich’s ataxia; MRI, magnetic resonance imaging; PET, positron emission tomography; RFC1, replication factor C subunit 1; SCA, spinocerebellar ataxia; VEMP, vestibular-evoked myogenic potentials; vHIT, video head impulse test; WGS, whole genome sequencing.

## CONCLUSION

The recognition of hereditary cerebellar ataxias is increasing and becoming frequent presentations to otolaryngology clinics. We have reviewed the common conditions that cause cerebellar ataxia and vestibular involvement. In the context of an acute presentation, it is worth considering AICA stroke and Wernicke’s encephalopathy. In patients with slowly progressive syndromes, it is worth considering hereditary ataxias, in particular SCA27B and CANVAS, with other SCAs and mitochondrial disorders also part of the differential diagnosis. Future studies using vestibular tests are crucial for better understanding the pathophysiology of vestibular involvement in cerebellar ataxia.

## ACKNOWLEDGMENTS

The authors acknowledge Sofia Mermelstein and Isaac Bocai for their kindness in providing some of the videos.

## Supplementary Material
