# Supplementary material for: Vestibular Loss and Cerebellar Ataxia: A Practical Approach
Source: Ear Hear. 2025 Aug 12;47(1):85–94. doi: 10.1097/AUD.0000000000001708 (PMC12700678; doi:10.1097/AUD.0000000000001708)
Supplement: Supplementary file 4 [file aud-47-085-s004.pdf]

Box 1. Clinical Case - CANVAS

A 56-year-old, female, Brazilian, married, one daughter, employed as a Physical Education teacher and right-handed. Approximately 15 years ago, she began experiencing intermittent imbalance, particularly during swimming lessons and while walking along the poolside. Initially, she attributed these episodes to cervical spine issues and stress. However, the symptoms have progressively worsened over time. Despite this gradual progression, the symptoms have not significantly interfered with her daily activities or professional duties. There have been no episodes of acute exacerbation, fluctuations, or identifiable triggers. She denies any leg weakness during this period but reports mild numbness and a tingling sensation in the fingertips of both her hands and feet. She denies vertigo symptoms, as well as hand tremors or coordination difficulties. Over the past six months, she has noticed that her speech has become "slurred." She denies any visual disturbances, including blurred or double vision, and reports no changes in her auditory function. For the past four years, she has experienced dysphagia for solids, accompanied by choking and significant coughing in the last year. There are no cognitive changes, and she remains independent in activities of daily living.

She has four siblings from the same parents. Her oldest brother exhibits similar symptoms, including gait incoordination and dysarthria, although she describes his condition as "worse than hers."

Neurological examination revealed gait ataxia (Video_4). Cerebellar examinations revealed clumsiness in the finger-to-nose test. Pin-prick and vibratory sensations were diminished in the lower limbs. A decreased sense of position on fingers and toes were observed. The bedside neurotological examination revealed dowbeating gaze-evoked nystagmus on lateral gaze (Video_5) and a bilaterally impaired head impulse test (Video_6), VVOR impairment (Video_2), smooth pursuit and saccades were performed without any significant abnormalities.

Laboratory and gene tests, nerve conduction study, pure tone audiometry, MRI and vHIT were requested. Laboratory examination showed no abnormality. Gene testing for spinocerebellar ataxia (SCA) types 3 and 6 was negative, and positive for CANVAS. Nerve conduction study showed absent sensory nerve action potentials in the upper and lower limbs bilaterally. Normal pure tone audiometry, and normal MRI. The vHIT (see below) presented bilaterally reduced VOR gains with the catch-up saccades in all semicircular canals.

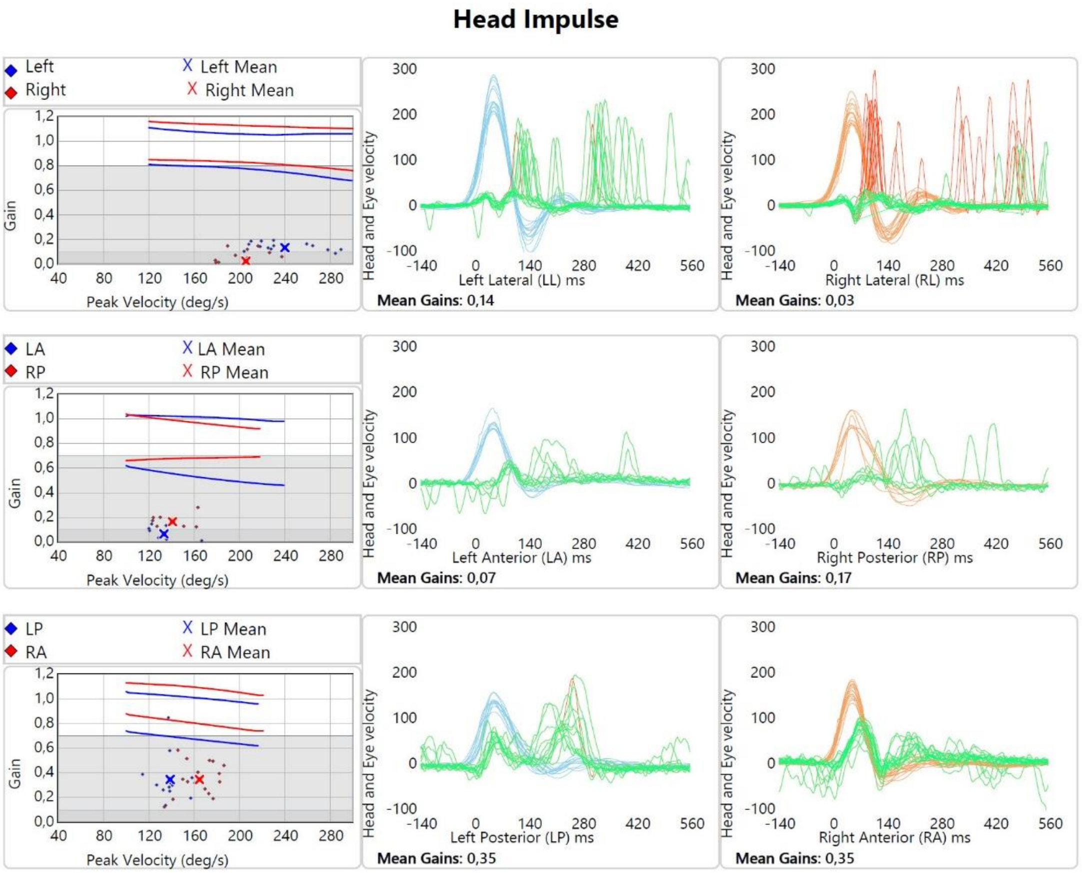

Video Head Impulse Test (vHIT) shows bilaterally reduced VOR gains, in all semicircular canals, with the catch-up saccades. Green and red (or blue) lines indicate the eye and head velocities (red to the right and blue to the left), respectively. Eye velocity is inverted.
